# From Pulses to Sleep Stages: Towards Optimized Sleep Classification Using Heart-Rate Variability

**DOI:** 10.3390/s23229077

**Published:** 2023-11-09

**Authors:** Pavlos I. Topalidis, Sebastian Baron, Dominik P. J. Heib, Esther-Sevil Eigl, Alexandra Hinterberger, Manuel Schabus

**Affiliations:** 1 Laboratory for Sleep, Cognition and Consciousness Research, Department of Psychology, Centre for Cognitive Neuroscience Salzburg (CCNS), Paris-Lodron University of Salzburg, 5020 Salzburg, Austria; pavlos.topalidis@plus.ac.at (P.I.T.); dominik.heib@proschlaf.eu (D.P.J.H.); esther-sevil.eigl@plus.ac.at (E.-S.E.);; 2 Department of Mathematics, Paris-Lodron University of Salzburg, 5020 Salzburg, Austria; 3 Department of Artificial Intelligence and Human Interfaces (AIHI), Paris-Lodron University of Salzburg, 5020 Salzburg, Austria; 4 Institut Proschlaf, 5020 Salzburg, Austria

**Keywords:** automatic sleep-staging, machine learning, CNN, wearables, hear-rate variability, inter-beat intervals

## Abstract

More and more people quantify their sleep using wearables and are becoming obsessed in their pursuit of optimal sleep (“orthosomnia”). However, it is criticized that many of these wearables are giving inaccurate feedback and can even lead to negative daytime consequences. Acknowledging these facts, we here optimize our previously suggested sleep classification procedure in a new sample of 136 self-reported poor sleepers to minimize erroneous classification during ambulatory sleep sensing. Firstly, we introduce an advanced interbeat-interval (IBI) quality control using a random forest method to account for wearable recordings in naturalistic and more noisy settings. We further aim to improve sleep classification by opting for a loss function model instead of the overall epoch-by-epoch accuracy to avoid model biases towards the majority class (i.e., “light sleep”). Using these implementations, we compare the classification performance between the optimized (loss function model) and the accuracy model. We use signals derived from PSG, one-channel ECG, and two consumer wearables: the ECG breast belt Polar® H10 (H10) and the Polar® Verity Sense (VS), an optical Photoplethysmography (PPG) heart-rate sensor. The results reveal a high overall accuracy for the loss function in ECG (86.3 %, κ = 0.79), as well as the H10 (84.4%, κ = 0.76), and VS (84.2%, κ = 0.75) sensors, with improvements in deep sleep and wake. In addition, the new optimized model displays moderate to high correlations and agreement with PSG on primary sleep parameters, while measures of reliability, expressed in intra-class correlations, suggest excellent reliability for most sleep parameters. Finally, it is demonstrated that the new model is still classifying sleep accurately in 4-classes in users taking heart-affecting and/or psychoactive medication, which can be considered a prerequisite in older individuals with or without common disorders. Further improving and validating automatic sleep stage classification algorithms based on signals from affordable wearables may resolve existing scepticism and open the door for such approaches in clinical practice.

## 1. Introduction

Because sleep is an important factor related to health, disease, overall quality of life and peak performance [1], more and more people are monitoring it using wearable devices. The pursuit of good sleep can sometimes go too far, creating an obsession with monitoring and quantifying sleep that can result in increased stress and discomfort [2], a condition termed orthosomnia (i.e., from the Greek “ortho” meaning straight or upright and the Latin “somnia”). Many users of wearables have been erroneously led to believe, however, that commercial wearables can accurately and reliably measure sleep, even though the majority lack scientific soundness and/or independent validation studies [3] against the gold standard, Polysomnography (PSG), which is a combination of electroencephalography (EEG), electrooculography, (ECG) and electromyography (EMG).

Erroneous feedback about one’s sleep (e.g., substantial underestimation of deep sleep, or wrong wake classification at sleep onset or during the night) can have serious adverse effects, exacerbating people’s misperception and even leading to negative daytime consequences [4]. Such erroneous feedback on wearables may also lead to inappropriate suggestions for adjusting sleep habits and work against the aim of promoting better sleep health [5]. This is particularly worrisome for people with sleep problems and preoccupations who are especially sensitive to feedback on their sleep [6,7,8]. The potential adverse effects of inaccurate feedback in combination with limited and pour validation studies against PSG certainly justify the skepticism around the broad use of wearables in the clinical field [9]. At the same time, there are undeniably benefits of using accurate wearable devices that affordably capture daily sleep changes in natural environments, outside of the laboratory. Especially in light of recent studies showing that implementing such technologies together with sleep intervention protocols can have positive therapy outcomes [10,11,12,13]. It is our opinion that such technologies, if optimized and carefully validated, will soon play a central role in research and clinical practice as they allow continuous sleep measurements (and feedback) in ecologically valid home environments and at an affordable cost.

However, only a few of the wearable technologies that claim multiclass epoch-by-epoch sleep classification have been transparent considering their sensing technology and models [14]. There are even fewer studies validated against PSG in suitable large (and heterogeneous) samples, using appropriate performance metrics (e.g., Cohes’s κ, sensitivity and specificity, F1) rather than solely relying on mean accuracy values per night or even overall epoch-by-epoch accuracy percentages across sleep stages (see Topalidis et al. [15]). Among the few, Chinoy et al. [16] recently compared the performance of seven consumer sleep-tracking devices to PSG and reported that the reviewed devices displayed poor detection of sleep stages compared to PSG, with consistent under- or overestimation of the amount of REM or deep sleep. Chee et al. [17] validated two widely used sensors, the Oura ring (v. 1.36.3) and Actiwatch (AW2 v. 6.0.9), against PSG in a sample of 53 participants with multiple recordings each, varying in length (i.e., 6.5 8 or 9 h). Compared to PSG, the Oura ring displayed an average underestimation of about 40 min for TST (Total Sleep Time), 16 min for REM (Rapid Eye Movement) sleep, and 66 min for light sleep, while it overestimated Wake After Sleep Onset (WASO) by about 38 min and deep (N3) sleep by 39 min. Altini and Kinnunen [18] examined 440 nights from 106 individuals wearing the Oura ring (v. Gen2M) and found a 4-class overall accuracy of 79% when including various autonomic nervous system signals, such as heart-beat variability, temperature, acceleration and circadian features. Crucially, the results of Altini and Kinnunen [18] suggest that it is the autonomic nervous system signals, such as Heart-Rate Variability (HRV), that drive higher accuracy of 4-class classification performance, as it is drastically increased when adding these signals into the model trained only on movement and temperature signals.

**Figure 1:**
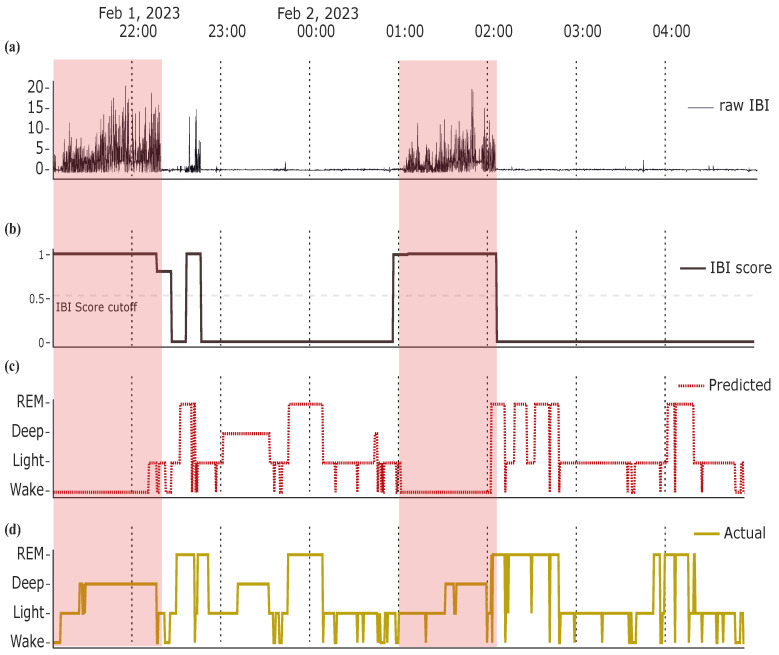
Bad IBI signal, if not detected, can lead to erroneous sleep stage classification. There are bad epochs in (**a**) the raw IBI signal that can be identified using (**b**) an advanced IBI signal quality control procedure based on a trained random forest model. Due to bad signal quality, sleep staging these epochs are erroneously classified (**c**), misrepresenting thus the ground truth (**d**).

We have recently developed a 4-class sleep stage classifications model (wake, light, deep, REM) that reaches up to 81% accuracy and a κ of 0.69 [15], when using only data from low-cost HRV sensors. Although such classification accuracies are approaching expert inter-rater reliability [15], there are edge cases where classification is particularly difficult (e.g., longer periods of rest and absent movement while reading as compared to falling asleep) and prone to errors, and may result in erroneous feedback to the users. To deal with these cases, we suggest first to implement advanced signal quality controls, that detect artefacts related to sensor detachment, or excessive movements during sleep that leads to insufficient signal quality (i.e., inter-beat interval estimations) for meaningful classification. In fact, some of the previously suggested models developed for one-channel ECG recordings (e.g., Mathunjwa et al. [19], Habib et al. [20], Sridhar et al. [21]) address such issues by implementing individual normalization and simple outlier corrections (e.g., linearly interpolating too short/long inter-beat intervals). However, we illustrate in Figure 1 an example from our data, that such a simple approach is sometimes insufficient, as bad IBI signal quality can lead to erroneous sleep stage classification, and we thus suggest incorporating advanced machine learning approaches for bad signal detection and more reliable classification.

**Figure 2:**
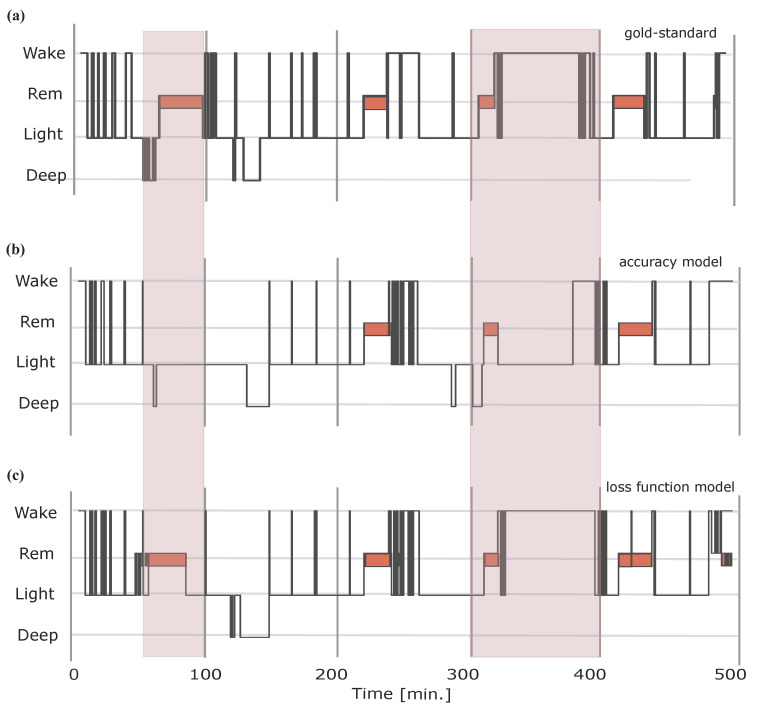
Example of a night where the accuracy model overestimates the majority class (light sleep). Panel (**a**) displays the actual PSG-based hypnogram as sleep staged automatically using the G3 sleepware gold-standard. Panel (**b**) displays sleep staging using the “accuracy model”, while panel (**c**) displays the “loss function model”. Note that the accuracy model displays a bias towards the majority class (e.g., epochs marked in red shading), as it strives to maximize overall classification accuracy, especially in cases where the model is unsure. In contrast, the loss function incorporates the skewed class distribution using categorical cross entropy weighted by class, thus correcting for a bias towards the majority class. In this example, both predicted hypnograms (**a**,**b**) use signals derived from the H10 sensor.

Additionally, to optimize sleep classification of edge cases we need to consider the skewness in the distribution of sleep phases across the night (i.e., a disproportionate amount of sleep stages across the night with light sleep dominating). This becomes evident when one acknowledges that algorithms can reach epoch-by-epoch classification accuracies of up to 65–70% when an algorithm simply classifies “light sleep” (N1 and N2) throughout the night. A model that is trained for optimal overall classification accuracy, such as the one suggested in Topalidis et al. [15], will display a bias towards the majority class (light sleep), resulting in poorer performance on less populated classes, such as wake and deep sleep. It is however crucial for the user that specific periods of waking and deep sleep are not misclassified as this substantially decreases the user’s trust in the sleep analysis and consequently any potential sleep intervention. We suggest a model that opts for the minimum weighted cross-entropy loss function value, that encapsulates the skewness of the sleep stage distribution, thus resulting in unbiased classification. Figure 2 illustrates the bias of the accuracy model towards the majority class and how opting for the loss function model resolves this issue.

We here explore the benefits of applying an IBI-correction procedure, as well as opting for the loss function in classifying sleep. We tested our updated model on a new sample of 136 subjects, whose sleep was recorded using ambulatory PSG, in their homes, for one or more nights. We are using an (i) ECG gold-standard (providing IBIs), (ii) a breast-worn belt measuring IBIs (Polar® H10) as well as (iii) a pulse-to-pulse interval device worn on the upper arm (Polar® Verity Sense-VS) as input signals. We put particular emphasis on the upper arm VS as it is a more comfortable sensor to sleep with than the H10 breast belt, without any notable compromise on sleep classification. Finally, we assess whether wearables such as the ones tested here are even accurate in classifying sleep in users with health issues and who, as a consequence, are taking central nervous system medication (psychoactive substances) and/or medication with expected effects on the heart (beta blockers, etc.).

## 2. Methods

### 2.1. Participant

We recorded ambulatory PSG from 136 participants (female = 40; Mean Age = 45.29, SD = 16.23, range = 20–76) who slept in their homes, for one or more nights. In total 265 nights of recordings including the gold standard PSG and ECG. From these participants, 112 wore a Polar® H10 chest sensor (see the Section 2.2) and 99 participants wore the Polar® Verity Sense, for 178 and 135 nights, respectively. This sample was part of an ongoing study, investigating the effects of online sleep training programs on participants with self-reported sleep complaints, which included participants with no self-reported acute mental or neurological disorders and capable of using smartphones. While we did not set strict exclusion criteria our sample naturally comes with people having sleep difficulties. Specifically, 84.8% of our sample had a Pittsburgh Sleep Quality Index (PSQI) score above 5, with an average score of 9.36 (SD = 3.21). For a subset of participants, we took a medical history and grouped them in those who were on psychoactive and/or heart medication (with medication, N = 17, Nnights = 40), and those who reported taking no medication (without medication, N = 39, Nnights = 87). The study was conducted according to the ethical guidelines of the Declaration of Helsinki.

### 2.2. Materials

We recorded PSG using the ambulatory varioport 16-channel EEG system (Becker Meditec®, Karlsruhe, Germany) with gold cup electrodes (Grass Technologies, Astro—Med GmbH®, Rodgau, Germany), according to the international 10–20 system, at frontal (F3, Fz, F4), central (C3, Cz, C4), parietal ( P3, P4) and occipital (O1, O2) derivations. We recorded EOG using two electrodes (placed 1 cm below the left outer canthus and 1 cm above the right outer canthus, respectively), and the chin muscle activity from two EMG electrodes. Importantly, we recorded the ECG signal with two electrodes that we placed below the right clavicle and on the left side below the pectoral muscle at the lower edge of the left rib cage. Before actual sleep staging, we used the BrainVision Analyzer 2.2 (Brain Products GmbH, Gilching, Germany) to re-reference the signal to the average mastoid signal, filter according to the American Academy of Sleep Medicine (AASM) for routine PSG recordings (AASM Standards and Guidelines Committee [22]) and downsampled to 128 Hz (the original sampling rate was at 512 Hz). Sleep was then automatically scored in 30-s epochs using standard AASM scoring criteria, as implemented in the Sleepware G3 software (Sleepware G3, Koniklijke Philips N.V., Eindhoven, The Netherlands). The G3 software is considered to be non-inferior to manual human staging and can be readily used without the need for manual adjustment [23]. All PSG recordings in the current analysis have been carefully manually inspected for severe non-physiological artifacts in the EEG, EMG as well as EOG, as such effects would render our PSG-based classification (serving as the gold standard) less reliable.

Having conducted extensive research on multiple sensors, we decided on two Polar® (Polar® Electro Oy, Kempele, Finland) sensors as they came with the most accurate signal as compared to gold-standard ECG [24,25,26]: the H10 chest strap (Model: 1W) and the Verity Sense (VS, Model: 4J). Apart from good signal quality, both sensors have good battery life (about 400 h for H10 and 30 h for VS Sense), and low overall weight and volume, which makes them comfortable to sleep with.

### 2.3. Missing Data Handling and Synchronization

For each home recording, sleep staging was computed using PSG and the G3 software (for details, see Section 2.2) that served as the gold standard. As the beginning and the end of PSG and wearable recordings were manually set and therefore do not perfectly overlap in time, inter-beat interval time series of the wearable sensors were synchronized in time to the inter-beat intervals from the ECG channels of the PSG recording (using a windowed cross-correlation approach before sleep classification). As recordings involved ambulatory PSG and sensor recordings in home settings, we excluded recordings in cases where (i) signal quality was poor (>25% bad IBI data), (ii) sensor data could not be read out, (iii) recordings were too short due to battery failure, and (iv) where synchronization between sensor and PSG was not possible (due to one of the two time series being too noisy). For eligible recordings with lower than 25% bad signal quality we “excluded” (and filled with padding) a total of 806 epochs, lasting 30 s, for ECG (which is 0.32% of all epochs), 3506 epochs for H10 (=2.1%), and 818 epochs for VS (=0.66%). In total, this resulted in 37 H10 and 54 VS recordings that were lost due to the aforementioned problems.

### 2.4. Model Optimization

Model (accuracy model) training has been described in Topalidis et al. [15]. We have further optimized this model by accounting for model biases towards the majority class (light sleep). We aim to oppose such biases even more by selecting the final model based on the loss function value, which already incorporates the skewed class distribution (loss function model). In contrast to the accuracy model, the optimized model includes gender and age as features to account for potential changes in sleep architecture [27]. In addition, we have implemented an IBI quality control procedure: we trained a random forest model on a subset of IBI windows, which were manually labeled in terms of good and bad IBI segments and calculated a set of IBI features on a fixed time window of 10 min that were used as input. We started with the feature set used by Radha et al. [28] but reduced it to a set of 7 features based on permutation feature importance values. Furthermore, we adjusted the threshold of the output value to account for the model’s ability to deal with minor levels of noise and distortion. The IBI quality control was applied after the actual sleep staging, and sleep stage labels of 30-s segments including bad IBIs were then replaced with the surrounding scorings of clean segments (i.e., segments without bad IBIs). In case more than 25% of all 30-s segments of a single night included bad IBIs, then the whole night was characterized as unstageable.

### 2.5. Sleep Parameters

We chose a few key sleep parameters extracted from PSG and the wearable sensors to explore their relationship and the agreement. We focused primarily on the following objective sleep variables: Sleep Onset Latency (SOL), defined as the time from lights out until the start of the first epoch of any stage of sleep (an epoch of N1, N2, N3, or R), Sleep Efficiency (SE: total sleep time/ time in bed × 100), Wake After Sleep Onset (WASO), Total Sleep Time (TST), as well as Deep and REM (Rapid Eye Movement) sleep measured in minutes.

### 2.6. Model Performance and Statistical Analysis

We used standard measures for estimating model performance, such as overall classification accuracy, Cohen’s κ [29], as well per-class recall and precision values, which are displayed using confusion matrices. Note that the performance metrics displayed in the confusion matrices are computed by averaging all aggregated epochs. We further explored the performance of each sensor using a Wilcoxon signed-rank test where data for both the gold standard and each sensor existed. In addition, we examined the performance of the two models for each sleep stage separately by computing the F1 score and comparing them with a one-tailed non-parametric Wilcoxon signed-rank test expecting higher F1 scores for the loss function model. A Wilcoxon signed-rank test was also used to compare the classification accuracies between the recordings of participants on psychoactive or heart medication and those with no medication, as well as to see how these two groups differ in age and PSQI scores.

To explore the relationship between the PSG and the two sensors on sleep parameters, we conducted Spearman’s Rank-Order correlation (denoted as ρ) and visualized the linear trends using scatterplots. We determined the agreement between the PSG and wearables sleep parameters using Bland–Altman plots, reporting the biases, Standard Measurement Error (SME), Limits of Agreement (LOA), Minimal Detectable Change [30] (MDC = $SME\cdot 1.96\cdot \sqrt{2})$, as well as absolute interclass correlation (ICC, a two-way model with agreement type [31]). The MDC, or else smallest detectable change, refers to the smallest change detected by a method that exceeds measurement error [30]. The intraclass correlation of reliability encapsulates both the degree of correlation and agreement between measurements [31]. According to Koo and Li [31], values less than 0.5, between 0.5 and 0.75, between 0.75 and 0.9, and greater than 0.90 are indicative of poor, moderate, good, and excellent reliability, respectively. All data and statistical analysis was performed in R (R Core Team [32]; version 4).

## 3. Results

### 3.1. Comparison of the Accuracy and Loss Function Models and Performance after Optimization

**Figure 3:**
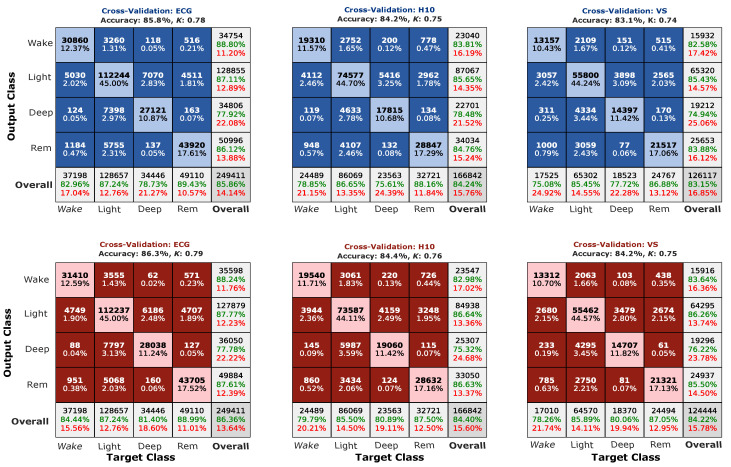
Confusion matrices of accuracy (**blue**) and loss function models (**red**). The IBIs were extracted from gold-standard ECG (**left**), chest-belt H10 (**middle**), and the PPG VS (**right**), and were classified using the two models. In each confusion matrix, rows represent predicted classes (Output Class) and columns represent true classes (Target Class). Cells on the diagonal indicate correct classifications, while off-diagonal cells represent incorrect classifications. Each cell displays the count and percentage of classifications. Precision (truePositives/(truePositives+falsePositives)) is displayed on the gray squares on the right, while recall (truePositives/(truePositives+falseNegatives)) is displayed at the bottom. The number of epochs has been equalized between the two models for a more fair comparison. Note that next to the small improvement in the overall accuracy compared to the accuracy model, the loss function model displays an increase in the recall of wake and deep sleep stages. This is arguably enough to address some of the nights that are difficult to classify.

**Figure 4:**
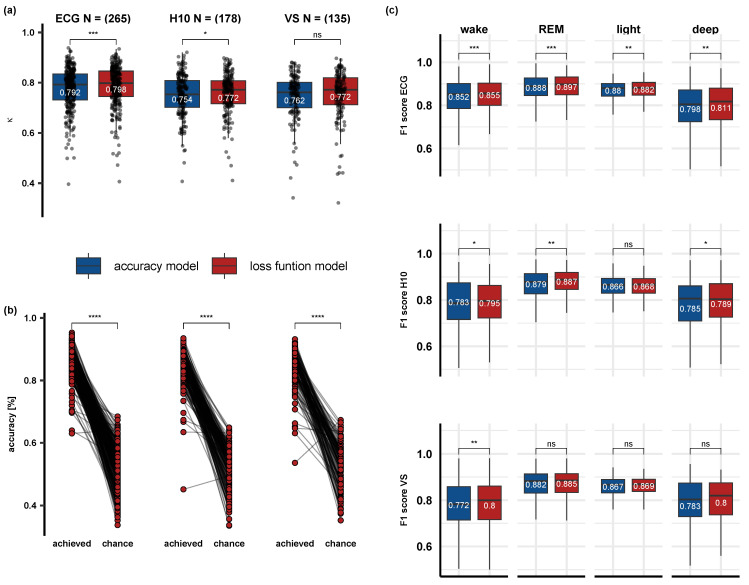
Performance metrics of the accuracy and loss function models as computed for ECG (**left**), H10 (**middle**), and VS (**right**). (**a**) The loss function model yielded a small but significant increase in the κ values for all sensors. (**b**) The loss function model displayed significant higher classification accuracies (**b**) compared to staging the majority class for the whole recording. (**c**) When considering the performance of the two models separately in each class, as reflected in the class F1 scores, we observed a small but significant increase in the performance of the loss function model. ns—not significant, <0.05 *, <0.01 **, <0.001 ***, <0.0001 ****.

Figure 3 illustrates the confusion matrices for each model and sensor. When looking at the overall performance, we did observe a small (in the magnitude of 1 to 2%) increase in the overall classification accuracy for the loss function model. Statistical within-subject comparison of the two models showed a benefit only for the ECG (*p* < 0.001) recordings, but not for the H10 (*p* = 0.094) and VS (*p* = 0.13). Comparison of κ values yield a significant increase for the ECG (*p* < 0.001) and H10 (*p* = 0.036), but not VS (*p* = 0.14). Figure 4a displays the average model κ values for each sensor separately. In addition, when using the loss function model, κ values for ECG (M = 0.798, SD = 0.08) were significantly higher (*p* < 0.001) than the H10 (M = 0.772, SD = 0.09) and VS (M = 0.772, SD = 0.1), while the two sensors did not significantly differ. Compared to classifying only light sleep for the whole recording, considered here as the chance level, the loss function model achieved significantly higher classifications accuracies (*p* < 0.001) for all sensors. Figure 4b illustrates the classification accuracies achieved for every recording, using the loss function model, and the corresponding accuracies when staging only the majority class. Finally, we tested whether F1 scores, incorporating both the precision and recall of the model, improve significantly using the loss function model when considering each class separately. When looking at the ECG sensor we observed a small but significant increase in the classification accuracies in all sleep stages (wake: *p* = 0.001; REM: *p* < 0.001; light: *p* = 0.004; deep: *p* = 0.003). F1 scores in H10 were also higher for the loss function in all sleep stages, except for light sleep (*p* = 0.56). Only wake sleep showed a benefit from the loss function model in VS (*p* = 0.015). The median F1 score for each sensor, model, and sleep stage are illustrated in Figure 4c.

### 3.2. Correlation and Agreement between the Gold-Standard PSG and the Two Wearable Devices on Primary Sleep Parameters

We observed significant (*p* < 0.001) high Spearman correlations between all PSG and VS derived sleep parameters of interest. More specifically, between H10 and PSG we found a high correlation in Sleep Onset Latency (ρ = 0.82, *p* < 0.001), Sleep efficiency (ρ = 0.88, *p* < 0.001), and Total Sleep Time (ρ = 0.96, *p* < 0.001), as well as, Wake After Sleep Onset (ρ = 0.89, *p* < 0.001), Deep (ρ = 0.6, *p* < 0.001), and REM sleep (ρ = 0.76, *p* < 0.001).

**Figure 5:**
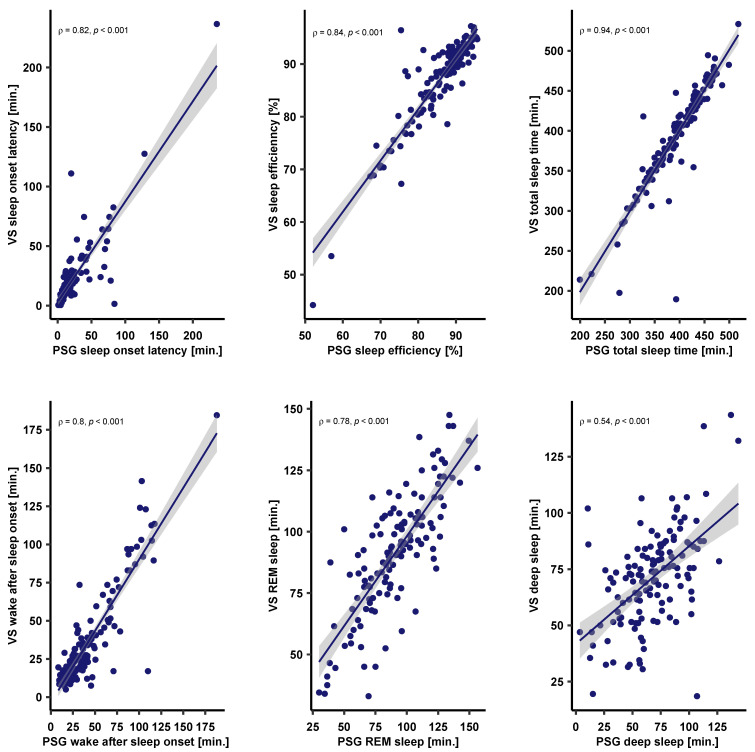
Correlations between VS and PSG sleep parameters as computed with Spearman’s rank correlations (ρ). PSG sleep parameters are plotted on the x’ axis and VS metrics are plotted on the y’ axis. The individual points reflect each recording. The solid line reflects the corresponding linear model and the shaded areas reflect the 95% confidence intervals. Note that all sleep metrics correlate highly with PSG, with deep sleep showing the weakest positive correlation.

Similar correlations were observed between VS and PSG: Sleep Onset Latency (ρ = 0.82, *p* < 0.001); Sleep efficiency (ρ = 0.84, *p* < 0.001), and Total Sleep Time (ρ = 0.94, *p* < 0.001); Wake After Sleep Onset (ρ = 0.8, *p* < 0.001); Deep (ρ = 0.6, *p* < 0.001); REM sleep (ρ = 0.78, *p* < 0.001). Figure 5 displays the correlation between VC and PSG.

**Figure 6:**
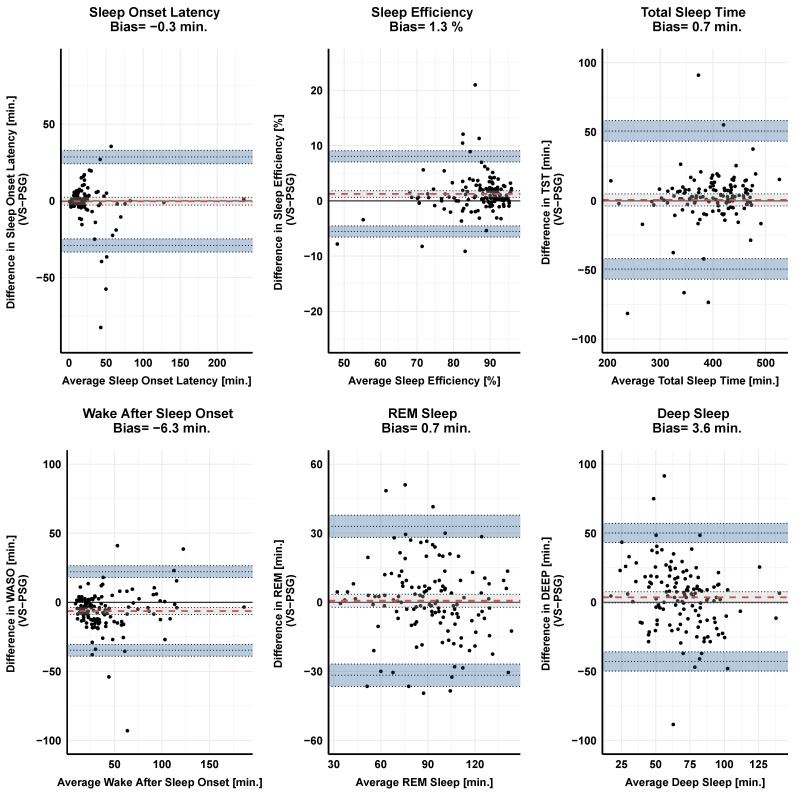
Agreement between the PSG-based sleep metrics and VS sensor, as visualized using Bland–Altman plots. The dashed red line represents the mean difference (i.e., bias) between the two measurements. The black solid line represents the point of equality (where the difference between the two devices is equal to 0), while the dotted lines represent the upper and lower limits of agreement (LOA). The shaded areas indicate the 95% confidence interval of bias and lower and upper agreement limits. A positive bias value indicates a VS overestimation, while a negative bias value reflects a VS underestimation, using the gold standard, PSG, as point of reference (VS-PSG). Note that VS underestimates SOL and WASO, while it overestimates the rest of the sleep parameters. However, the degree of bias here is minimal.

We explored the extent of the agreement between the gold standard and both sensors using Bland–Altman plots on the sleep parameters of interest. Table 1 and Table 2 summarize the extent of agreement between the gold standard, PSG, and the two wearable devices, H10 and VS on the sleep parameters of interest. In Figure 6 we visualize the agreement between PSG and VS using Bland–Altman plots.

### 3.3. Effects of Psychoactive and/or Heart Affecting Medication on Classification Performance

**Figure 7:**
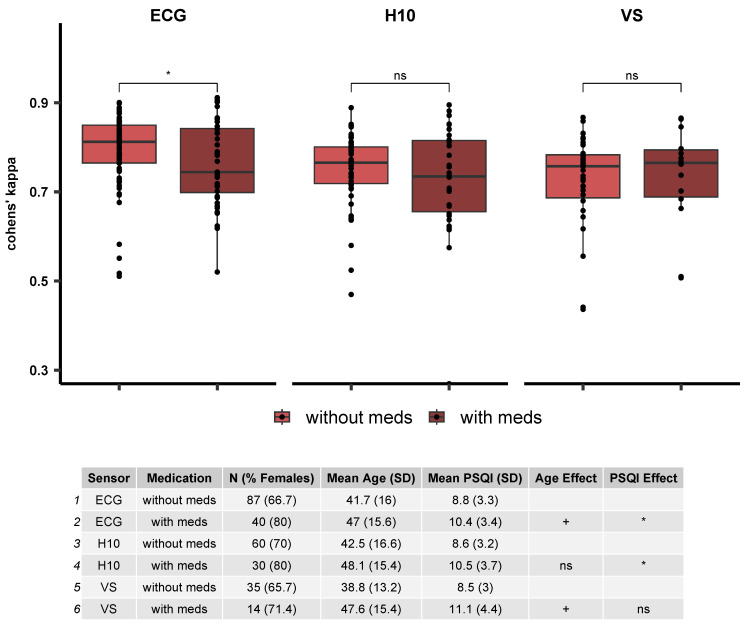
The effects of heart or psychoactive medication on sleep stage classification using the optimized model. The boxplot illustrates the median κ values between recordings obtained from people with and without medication, separately for each sensor. The table contains the group descriptives including the number of subjects, age and PSQI group averages, as well as the statistical effects of Age and PSQI. Note that there are significant differences between the two groups in the ECG recordings, but at the same time a trend for age and a significant difference in the PSQI scores. ns—not significant, <0.05 *.

We explored how medication can influence the classification performance by comparing the performance metrics, accuracy and κ, between the recordings of participants with and without medication. We found significant effects of medication for the ECG recordings (*p* = 0.033), but such a difference did not reach statistical significance for the H10 (*p* = 0.52) and VS (*p* = 0.87). The same effects were observed when using accuracy as a performance metric. Note that there was a trend for age differences in ECG and VS recordings (ECG: *p* = 0.06; VS: *p* = 0.06), while the two groups also differ significantly in their PSQI scores for the ECG (*p* = 0.023) and H10 (*p* = 0.027), but not the VS recordings. Figure 7 illustrates differences in κ values between the two groups for each sensor.

## 4. Discussion

In the current study, we validated our previous findings in a new sample and showed that the model suggested in the study of Topalidis et al. [15] can be optimized further by including advanced IBI signal control and opting for the loss function for choosing the final classification model. Results reveal statistically higher classification performance for the loss function model, compared to choosing the model with the highest overall accuracy during validation (accuracy model), although numerically the benefit is small. We discuss why even a small but reliable increase is relevant here while highlighting that we optimized a model that already displays very high classification performance. We further show that psychoactive and/or heart-affecting medication does not have a strong impact on sleep stage classification accuracy. Lastly, we evaluated our new optimized model for measuring sleep-related parameters and found that our model shows substantial agreement and reliability with PSG-extracted sleep parameters.

In the following section, we discuss the results, acknowledge the limitations of the current study, and provide an outlook on sleep classification using wearables in sleep research and clinical practice for the near future.

We have reasoned that opting for the loss function model will have a beneficial effect on classification performance, minimizing biases towards the majority class. When looking at overall performance in the confusion matrices (Figure 3), we descriptively observed a small increase in the overall classification accuracy for the loss function model. Such a difference was significant when comparing the κ values within-subject, as shown in Figure 4a, without epoch aggregation, only for the ECG, but not the H10 and VS. As the effect size of the improvement is rather low (0.06% improvement for the ECG), this could reflect a mere statistical effect, reflected in differences in the number of available data among the sensors. Furthermore, a comparison between the sensors showed that the ECG performance, regardless of performance metric (i.e., κ or accuracy) displayed a better performance for the ECG compared to the two wearables (app. 0.02 κ). This suggests that better signal quality and less variability in classification performance when using the ECG could lead to more accurate classification.

In addition, we observed that the optimized model performs significantly better than a model that would only predict the majority class (light sleep) for the entire recording. Such comparison is necessary, as it is possible to reach 70% classification accuracy for some nights when sleep staging only the majority class (cf. Figure 3). When comparing the two models statistically per class using F1 scores extracted per recording, we observed a clear benefit using the loss function model in all sleep stages when looking at ECG recordings. Although the increase in F1 scores for the accuracy model is numerically small (ranging from 0.02 to 0.09), it is clear that opting for the loss function leads to classification improvements. Such an increase in F1 scores is most likely due to improvement in the model recall, as precision did not change with model optimization (cf. Figure 3, ECG). In H10 recordings, we observed benefits for wake, REM, and deep sleep, while the VS we observed a benefit only for wake. Similarly to the ECG sensors, these improvements in F1 score for the loss function model stem primarily from a boost in the recall of the model rather than the precision. The absence of significant benefits for deep sleep in the VS sensors could be explained by higher variability and smaller sample sizes compared to the ECG.

Although one could discuss whether such small performance benefits are meaningful, the classification accuracies reported here approach human inter-rater agreement. Therefore any slight increase in classification performance could contribute to dealing with edge cases or nights that are difficult to classify. As pointed out in Topalidis et al. [15] it is estimated that at experts display an agreement of around 88% for four classes, and compared to the 84% (κ≈ 0.75 ) on average in our study across wearable devices this equals to 95.45% agreement between the current model and PSG-based human scorings. Importantly, the observed benefits in wake and deep sleep classification are crucial as they cause the most irritation in the end-user if experienced as incorrect.

Furthermore, we evaluated the agreement and reliability of our model against PSG on primary sleep parameters. Particular emphasis was put on the VS sensor, as it is the more comfortable sensor to sleep with, while at the same time providing an indirect measure of heart-rate variability using the pulse-to-pulse intervals of the PPG. Surprisingly, only for a few sleep variables, the ECG-based H10 was found to be better in accuracy. For all stages apart from deep sleep (0.5), the key sleep parameters showed high correlations (0.7 to 0.94) between PSG and VS. The highest correlation was found for total sleep time and showed almost perfect agreement (r = 0.94). These correlational analyses were nicely complemented by intra-class correlations, which likewise indicated moderate (for deep sleep) to excellent reliability. Systematic bias of the VS against the PSG gold standard was visualized using Bland–Altman plots and was found to be minimal. Keeping in mind that if the end-user has a sleep issue, “deep sleep” seems to be a crucial measure, as it has been related to physical and mental recovery, immune system strengthening, or the disposal of brain waste products via the glymphatic system (e.g., Reddy and van der Werf [33]). We also provided the MDC metric, which indicates the smallest change detected by a method that exceeds the measurement error [30], and found that with both the H10 and VS there is a very good resolution that is accurate up to ±5 min across sleep parameters. Following the results reported in the current study, future emphasis shall thus be put on further increasing the classification accuracy for the “deep sleep” class, while implementing standardized frameworks for evaluating the performance of sleep-tracking technology [34].

The classifications reported here, are to our knowledge among the highest classification performances using wearable devices, while capitalizing only on a single source of signal, namely the IBI signal. The classification approach here takes into consideration recommendations for optimal model training such as incorporating the temporal dynamics of sleep (information about epoch N is contained in epochs N − 1 and N + 1). Ebrahimi and Alizadeh [35], for example, systematically reviewed automatic sleep staging using cardiorespiratory signals, and suggested that by taking into account signal delays and implementing sequence learning models that are capable of incorporating temporal relations results in better classification performance.

We also explored the effects of psychoactive and heart medication on sleep stage classification performance. We reasoned that such medication could have a direct effect on PSG and ECG [36,37] and thereby affect relevant features of the signal, resulting in decreased classification performance. We observed a small but statistically significant decrease in κ values in people with and without medications only for the ECG data, but not the wearables. These results could be explained by the large difference in available data between the devices in combination with small effect sizes. It is important to note, however, that for the ECG data age and PSQI differences were observed between the two groups (the group with medication being older and having worse sleep quality) and thus the drop in classification performance could be driven by these differences. However, the median κ for all classified recordings from participants on medication groups was above 0.07 for all devices, suggesting a substantial agreement in people taking psychoactive and/or heart-affecting medication. Future studies should look at the effects of psychoactive and/or heart-affecting medication, or even stimulants (e.g., caffeine), on sleep staging more closely, including more controlled or even within-subjects designs, to explore how even subtle changes in the cardiac signal can impact sleep stage classification.

As Western health systems increasingly adopt digital health interventions including sleep (see Arroyo and Zawadzki [38] for a systematic review on mHealth interventions in sleep), objective and reliable tracking of the effects of such interventions becomes more and more relevant and allows for ecologically valid and continuous measurements [9] in natural home settings. Recently, for example, Spina et al. [11] used sensor-based sleep feedback in a sample of 103 participants suffering from sleep disturbances and found that such sensor-based sleep feedback can already reduce some of the insomnia symptoms. Interestingly, such feedback alone was however not enough to induce changes in the sleep–wake misperception, which may need additional interventions (see Hinterberger et al. [39]). Given that people with sleep problems and preoccupations about their sleep are especially sensitive to such feedback, there is a high ethical necessity to only provide certain and accurate feedback to patients to prevent negative side effects [4].

## Figures and Tables

**Table 1:** Table of Agreement between PSG and H10 sensor. Agreement between the gold standard PSG and H10 wearable in measuring the sleep parameters of interest. Note that there is good to excellent reliability on all sleep parameters, but deep sleep shows a moderate reliability. SOL = Sleep Onset Latency, SE = Sleep Efficiency, TST = Total Sleep Time, WASO = Wake After Sleep Onset, REM = Rapid Eye Movement Sleep, LOA = Limits of Agreement, upper–lower; SME = Standard Measurement Error; MDC = Minimal Detectable Change; ICC = Intra-Class Correlation.

Parameter	Mean PSG (SD)	Mean H10 (SD)	LOA	Bias	SME	MDC	ICC
SO [min.]	18.4 (18)	18.3 (21.6)	31.8 − 31.9	−0.1	1.2	3.4	0.7
SE [%]	85.5 (8.8)	86.3 (8.8)	10 −8.5	0.8	0.4	1.0	0.9
TST [min.]	400.4 (59)	402.5 (60.6)	49.2 −45	2.1	1.8	5.0	0.9
WASO [min.]	51.3 (42.3)	47.4 (40.9)	25.1 −32.9	−3.9	1.1	3.1	0.9
REM [min.]	92.2 (27.9)	92.9 (26.7)	34.9 −33.5	0.7	1.3	3.6	0.8
Deep [min.]	66.6 (28.3)	71.4 (23.4)	48.3 −38.8	4.8	1.7	4.6	0.6

**Table 2:** Table of Agreement between PSG and VS sensor. Agreement between the gold standard PSG and VS wearable in measuring the sleep parameters of interest. Similarly to H10, there is good to excellent reliability on all sleep parameters, but deep sleep shows moderate reliability. SOL = Sleep Onset Latency, SE = Sleep Efficiency, TST = Total Sleep Time, WASO = Wake After Sleep Onset, REM = Rapid Eye Movement Sleep, LOA = Limits of Agreement, upper–lower; SME = Standard Measurement Error; MDC = Minimal Detectable Change; ICC = Intra-Class Correlation.

Parameter	Mean PSG (SD)	Mean VS (SD)	LOA	Bias	SME	MDC	ICC
SO [min.]	22 (27.8)	21.8 (27.3)	28.6 −29.1	−0.3	1.3	3.5	0.9
SE [%]	86 (7.8)	87.3 (8.3)	8.1 −5.5	1.3	0.3	0.8	0.9
TST [min.]	397.6 (56)	398.3 (62)	50.6 −49.3	0.7	2.2	6.1	0.9
WASO [min.]	43.9 (30.5)	37.6 (32)	22.2 −34.8	−6.3	1.3	3.5	0.9
REM [min.]	90.7 (26.6)	91.4 (24.5)	33 −31.7	0.7	1.4	4.0	0.8
Deep [min.]	67.4 (27.3)	71.1 (21.6)	50.2 −42.9	3.6	2.1	5.7	0.5

## Data Availability

The data that support the findings of this study are available from the University of Salzburg. Restrictions apply to the availability of these data, which were used under license for this study.

## Abbreviations

The following abbreviations are used in this manuscript:

IBI	Inter-Beat-Interval
HRV	Heart Rate Variability
PSG	Polysomnography
ECG	Electrocardiography
